# Influenza Induces Lung Lymphangiogenesis Independent of YAP/TAZ Activity in Lymphatic Endothelial Cells

**DOI:** 10.21203/rs.3.rs-3951689/v1

**Published:** 2024-02-27

**Authors:** Erin Crossey, Senegal Carty, Fengzhi Shao, Jhonatan Henao-Vasquez, Alexandra B. Ysasi, Michelle Zeng, Anne Hinds, Ming Lo, Andrew Tilston-Lunel, Xaralabos Varelas, Matthew R. Jones, Alan Fine

**Affiliations:** Boston University Chobanian and Avedisian School of Medicine; Boston University Chobanian and Avedisian School of Medicine; Boston University Chobanian and Avedisian School of Medicine; Boston University Chobanian and Avedisian School of Medicine; Boston University Chobanian and Avedisian School of Medicine; Boston University Chobanian and Avedisian School of Medicine; Boston University Chobanian and Avedisian School of Medicine; Boston University Chobanian and Avedisian School of Medicine; Boston University Chobanian and Avedisian School of Medicine; Boston University Chobanian and Avedisian School of Medicine; Boston University Chobanian and Avedisian School of Medicine; Boston University Chobanian and Avedisian School of Medicine

## Abstract

The lymphatic system consists of a vessel network lined by specialized lymphatic endothelial cells (LECs) that are responsible for tissue fluid homeostasis and immune cell trafficking. The mechanisms for organ-specific LEC responses to environmental cues are not well understood. We found robust lymphangiogenesis during influenza A virus infection in the adult mouse lung. We show that the number of LECs increases 2-fold at 7 days post-influenza infection (dpi) and 3-fold at 21 dpi, and that lymphangiogenesis is preceded by lymphatic dilation. We also show that the expanded lymphatic network enhances fluid drainage to mediastinal lymph nodes. Using EdU labeling, we found that a significantly higher number of pulmonary LECs are proliferating at 7 dpi compared to LECs in homeostatic conditions. Lineage tracing during influenza indicates that new pulmonary LECs are derived from preexisting LECs rather than non-LEC progenitors. Lastly, using a conditional LEC-specific YAP/TAZ knockout model, we established that lymphangiogenesis, fluid transport and the immune response to influenza are independent of YAP/TAZ activity in LECs. These findings were unexpected, as they indicate that YAP/TAZ signaling is not crucial for these processes.

## Introduction

The pulmonary lymphatic vasculature plays a number of crucial roles in orchestrating the response to infection and tissue injury (1). These vessels are lined with specialized lymphatic endothelial cells (LECs), which maintain their identity through the constitutive expression of Prospero homeobox protein 1 (PROX1) (2) and are characterized by vascular endothelial growth factor receptor 3 (VEGFR3) expression (3–5). Lymphatics modulate interstitial fluid drainage and transport immune cells and antigen to lymph nodes (6). Signaling between LECs and immune cells also directs chemotaxis (7) and immune cell proliferation (8). During respiratory tract inflammation, these functions are essential, as excessive tissue fluid buildup or an ineffective immune response could severely compromise lung function.

New lymphatic vessel growth, or lymphangiogenesis, can be observed in inflamed tissue (9). Although this has been shown in the respiratory system in response to several types of injury, including bacterial infection (10, 11) and pulmonary fibrosis (12, 13), it is unknown whether it occurs in response to acute viral infections such as influenza.

Influenza viruses target the respiratory epithelium, triggering severe inflammation (14). In spite of advances in prophylaxis and treatment approaches, influenza infection is associated with 14% of acute respiratory disease-related hospitalizations (15) and an estimated 290,000–650,000 annual respiratory deaths worldwide (16). Concentrating on the host’s response, which is pivotal in determining influenza’s severity and outcome, opens avenues for novel therapeutic interventions. In this context, lymphatic vessel dysfunction might weaken tissue resilience and impede pathogen removal. Facilitating pulmonary lymphatic responses could theoretically, therefore, enhance viral elimination, counteract damaging immune dysregulation and strengthen fluid removal in severe cases. The Hippo pathway, known for its high conservation and central role in cell proliferation, organ development and morphogenesis, and tissue responses to injury could be key in guiding lymphatic endothelial cell behavior during influenza infection (17–19).

Mechanical stimuli, such as stretch and shear forces are common environmental cues that modulate Hippo signaling (17, 18, 20, 21). In Hippo signaling, two downstream effectors jointly regulate gene expression: Yes-associated protein (YAP), encoded by the *Yap1* gene, and Transcriptional co-activator with PDZ-binding motif (TAZ), encoded by the *Wwtr1* gene. YAP and TAZ protein activity depend on intracellular localization. Nuclear YAP and TAZ regulate a wide variety of target genes, whereas cytoplasmic YAP and TAZ are targeted for degradation (22). Notably, YAP/TAZ depletion (23, 24) or hyperactivation (23) in LECs during embryonic development results in structurally aberrant and poorly functional lymphatics and lethality, highlighting its importance in early development of the lymphatic system. In adults, however, intact Hippo signaling in LECs is less critical for maintaining lymphatic integrity during homeostasis (23). The functional role(s) of Hippo signaling in adult lung LECs, particularly during inflammatory responses, remain unexplored.

In blood endothelial cells, mechanosensitive signaling (20, 21) and molecular signals such as vascular endothelial growth factor A (VEGF-A) (25–27) act through the Hippo pathway to regulate cell proliferation. The major pro-lymphangiogenic growth factor, VEGF-C, also influences Hippo signaling (23,24). While increased fluid influx and altered levels of cytokines and growth factors are key characteristics of the lung’s inflammatory response, to our knowledge, no study has investigated what role YAP/TAZ signaling might play in pulmonary lymphatic vessels as they respond to influenza-induced inflammation.

Here we show that the pulmonary lymphatic system undergoes extensive dilation and lymphangiogenesis in a mouse model of influenza infection. We show by lineage tracing that the expanded lymphatic network in the lung derives from existing LECs. We also demonstrate that there is a significantly higher number of proliferating pulmonary LECs during influenza. The resulting lymphatic network exhibits increased fluid drainage from the lung to mediastinal lymph nodes. In view of these findings, we investigated the role of the Hippo signaling pathway in LECs during influenza. Our results demonstrate that LEC-specific deletion of these Hippo pathway effectors revealed that YAP/TAZ signaling is dispensable for pulmonary lymphangiogenesis, lymphatic drainage and host response in the adult lung during influenza. These findings were unanticipated, as they suggest that Hippo-dependent signaling is not crucial for these processes in adults. This discovery yields new avenues for understanding the mechanisms underlying pulmonary responses to influenza A infection and the regulation of lymphatic function in adult lungs.

## Results

### Pulmonary lymphangiogenesis in influenza pneumonia.

**Figure 1:**
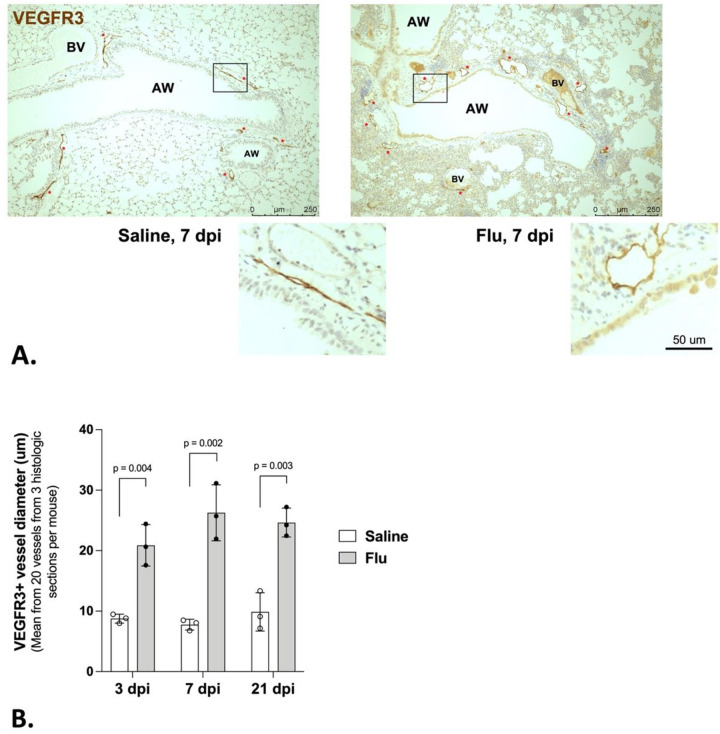
Lymphatic vessels dilate during influenza infection. (A) VEGFR3-positive lymphatic vessels (brown; also denoted with red stars) visualized via IHC are more dilated in influenza-infected lungs than in controls. (B) For each mouse, 20 lymphatic vessel diameters were measured using 3 separate histologic sections; mean and SD are shown for n = 3 mice per condition per time point (3-, 7-, and 21-dpi). Statistical significance by unpaired two tailed t-test with Welch’s correction.

To investigate the pulmonary lymphatic vessel responses to influenza infection, we intratracheally infected the left lobe of mice with PR8 influenza and harvested left lungs at 3-, 7- and 21-days post-infection (dpi) for paraffin-embedding and sectioning. Mice were weighed daily during the time-course of infection to assess morbidity (Supp. Figure 1). We then stained for VEGFR3 in lung sections of influenza-infected and control mice to identify lymphatic vessels. We observed a significant and sustained enlargement in the diameter of VEGFR3-positive vessels in the lung during influenza infection as soon as 3 dpi and until at least 21 dpi (Fig. 1a and b). This indicated that the lymphatic vessels are dilated in response to influenza infection.

**Figure 2:**
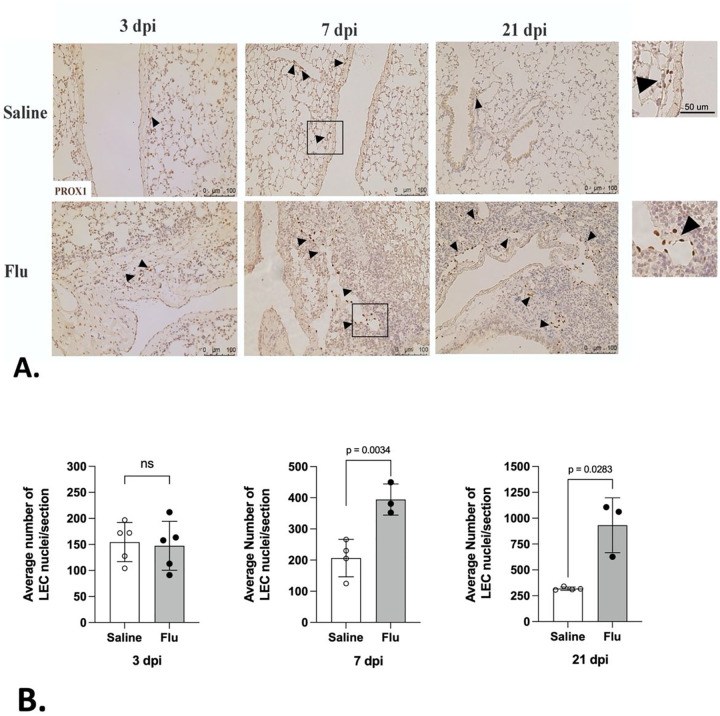
Influenza infection induces pulmonary lymphangiogenesis. (A) PRoX1-positive LEC nuclei (brown; also denoted with black arrowheads) visualized via IHC are more numerous in influenza-infected lungs than in controls at 7 and 21 dpi. (B) For each mouse, LEC nuclei were quantified using 3–5 tissue sections from influenza-infected and control mice; mean and SD are shown for n = 3–5 mice per condition per time point. Statistical significance by unpaired one tailed t-test with Welch’s correction.

To determine whether lymphangiogenesis accompanied vessel dilation, we quantified the lung LEC population by labeling LEC nuclei during a time-course of influenza infection. In order to do this, we stained for PROX1 at 3, 7 and 21 dpi and enumerated LECs in each histological section comparing influenza-infected lungs with controls. This experiment revealed a doubling of LECs by 7 dpi that continued to increase through 21 dpi (Fig. 2a and b). Our findings indicate that influenza infection not only leads to the enlargement of existing lymphatic vessels but also stimulates the expansion of LEC number.

### Pulmonary lymphangiogenesis in influenza is driven by LEC proliferation.

To identify the source of new LECs in the lymphatic network in influenza-infected lungs, we employed two independent approaches. Firstly, to label nascent DNA incorporation, 5-Ethynyl-2-deoxyuridine (EdU) was administered to mice during influenza infection. Lungs were harvested at 7 dpi and immunoflfluorescently co-stained for PROX1 and EdU (Fig. 3a and b). While proliferating LECs were very rarely observed in control tissues, approximately 20% of LECs in influenza-infected lungs had incorporated EdU by 7 dpi (3b). Secondly, we investigated the possibility that an exogenous progenitor cell might play a role in influenza-induced lymphangiogenesis. To address this question, tamoxifen-induced LEC lineage labeling was performed in PROX1-CreER^T2^/tdTomato mice prior to influenza infection. The proportion of tdTomato positive LECs observed during influenza infection was then compared to control lungs. Despite the increased number of LECs observed during influenza (data not shown), there was a similar proportion of lineage labeled cells at 7 dpi (Fig. 3c and d). These findings demonstrate that PROX1-negative progenitor cells are not contributing to lymphangiogenesis during influenza. However, we cannot exclude the possibility of a PROX1-positive progenitor. Notably, EdU uptake in LECs was not observed in the liver, heart, or esophageal tissues, indicating that LEC proliferation during influenza was specific to the lungs (data not shown). Taken together, these results suggest endogenous lung LEC proliferation in response to influenza infection contributes to LEC expansion.

### Lymphatic transport in influenza pneumonia.

**Figure 4:**
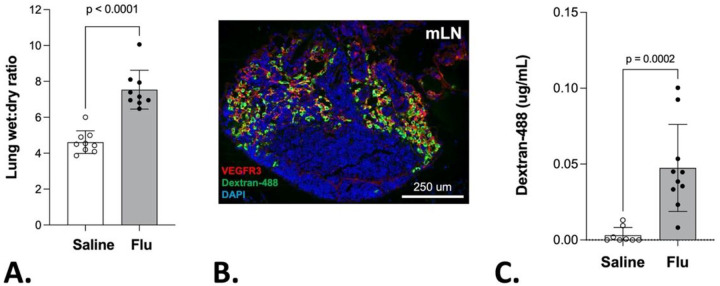
Lymphatic fluid drainage from the lung is enhanced during influenza infection. (A) Lung wet to dry weight ratios were obtained at 7 dpi from C57Bl/6 mice administered saline or PR8; mean and SD are shown for n = 9 mice per condition. Statistical significance by unpaired two-tailed Mann Whitney test. (B) Example histologic section of a mediastinal LN stained for VEGFR3 (red) and Dextran-488 (green). (C) Fluorometric quantification of Dextran-488 in mediastinal LN 15 minutes after instillation into left lung lobe. Mean and SD are shown for n = 8–10 mice per condition. Statistical significance by unpaired two-tailed Mann Whitney test.

Consistent with published literature, influenza infection leads to pronounced inflammation, and pulmonary edema. In this regard, we observed significantly higher lung wet-to-dry weight ratios during influenza infection as compared to controls, indicative of pulmonary edema (Fig. 4a). To investigate the functional properties of the expanded lymphatic network, we instilled a 10 kDa flfluorescent dextran molecule into the left lung airspaces and measured fluorescence in the lung-draining mediastinal lymph node (mLN) (Fig. 4b and c). We observed a significantly higher flfluorescent signal in the mLNs of influenza-infected mice as compared to control at 15 minutes. There was minimal flfluorescent signal detected in non-draining inguinal LNs indicating local lymphatic drainage as opposed to blood vessel drainage was primarily responsible for transport of dextran (data not shown). Collectively, these findings indicate that the expanded lymphatic network in the influenza-infected lung is associated with enhanced lymphatic transport.

### Role of Hippo signaling during influenza-induced pulmonary lymphangiogenesis.

The Hippo pathway is a fundamental mediator of cell proliferation during development and is activated in response to injury and mechanical cues. Notably, Hippo signaling in LECs is critical for lymphangiogenesis and lymphatic patterning during embryonic development (23, 24). To determine the role for Hippo signaling in LECs during influenza-induced lymphangiogenesis, we utilized a previously published model of Hippo pathway deletion in LECs (*Prox1-CreERT2/Yap1(YAP)- fl/Wwtr1(TAZ)*- fl, hereafter YAP/TAZ^△LEC^). In all experiments, Cre(−) littermates given tamoxifen were used as controls.

**Figure 5:**
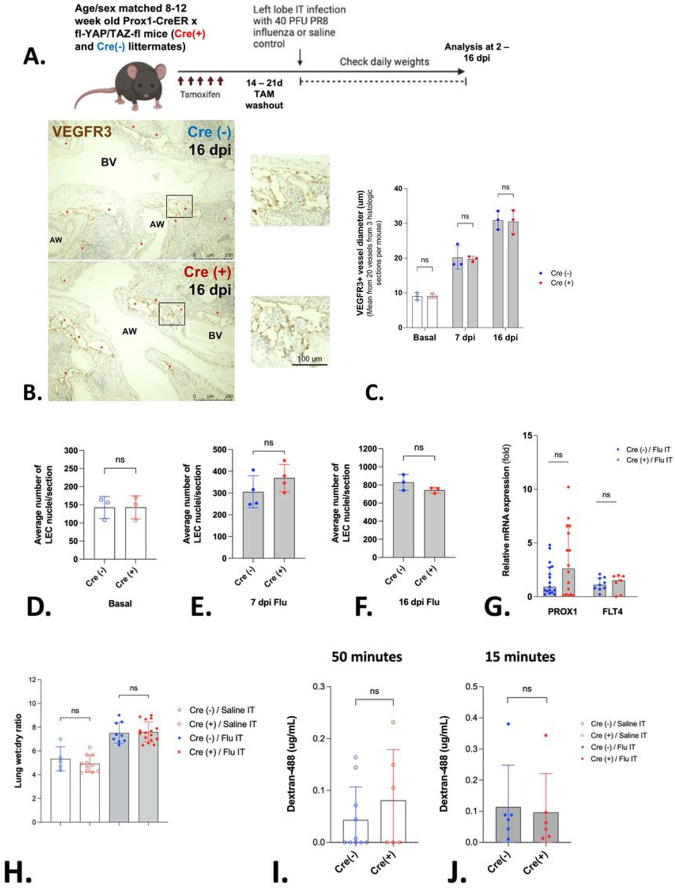
There is no significant difference in lymphatic histologic scoring or lymphatic fluid drainage from the lung between Cre(−) and Cre(+) YAP/TAZ^△LEC^ littermates at baseline or during influenza. (A) Experimental design; image created in BioRender. (B–C) VEGFR3-positive lymphatic vessels (brown, also denoted with red stars; airway, AW; blood vessel, BV) visualized via IHC in Cre(−) or Cre(+) littermates had similar diameters at baseline and during influenza at 7 or 16 dpi. For each mouse, 20 lymphatic vessel diameters were measured using 3 separate histologic sections; mean and SD are shown for n = 3 mice per condition per time point. Statistical significance by unpaired two-tailed Mann Whitney test. (D–F) PRoX1-positive LEC nuclei (brown) were visualized via IHC at baseline or during influenza at 7 or 16 dpi for Cre(−) and Cre(+) littermates. For each mouse, LEC nuclei were quantified using 3 tissue sections from influenza-infected and control mice; mean and SD are shown for n = 3 mice per condition per time point. Statistical significance by unpaired two tailed t-test with Welch’s correction. (G) Prox1 and Flt4 mRNA was quantified from left lobes collected from Cre(−) or Cre(+) littermates at 7 dpi after influenza infection. Median and IQR are shown for n = 7–17 mice per group. Statistical significance by unpaired two-tailed Mann Whitney test. (H) Lung wet to dry weight ratios were obtained at 7 dpi from Cre(−) or Cre(+) littermates administered saline or PR8 IT instillations; mean and SD are shown for n = 5–16 mice per condition per timepoint. Statistical significance by unpaired two-tailed Mann Whitney test. (I–J) Fluorometric quantification of Dextran-488 in mediastinal LN 50 minutes (saline) or 15 minutes (influenza) after IT instillation into left lung lobe of Cre(−) or Cre(+) littermates. Mean and SD are shown for n = 3–10 mice per condition per timepoint. Statistical significance by unpaired two-tailed Mann Whitney test.

First, we validated YAP and TAZ depletion in LECs using fluorescent staining of lung histologic sections and by validating the presence of the floxed YAP and TAZ alleles after tamoxifen administration (Supp. Figures 2 and 3). To confirm efficient PROX1-Cre-mediated recombination, we analyzed tdTomato expression in LECs in mice at least 2 weeks after tamoxifen administration to Prox1-CreER^T2^ / TdTomato mice (Supp. Figure 4). Next, we analyzed influenza-infected lungs for differences in histologic lymphatic phenotype, including lymphatic vessel diameter measurement and LEC enumeration as previously described. We identified no significant differences in these two parameters between Cre(+) and Cre(−) YAP/TAZ^△LEC^ littermates, either at baseline, 7 or 16 dpi (Fig. 5a–f). Similarly, no differences in *Prox1* or *Flt4* mRNA were observed in whole lung homogenates obtained from either Cre(+) or Cre(−) YAP/TAZ^△LEC^ littermates at 7 dpi (Fig. 5g).

To determine whether there were differences in pulmonary edema in the context of Hippo deletions, we measured lung wet-to-dry weight ratios during influenza as well as control conditions, and found no difference between Cre(+) and Cre(−) YAP/TAZ^△LEC^ littermates (Fig. 5h).

The dextran transport assay was utilized to interrogate the functionality of lymphatics for passive drainage in uninfected lungs. In these experiments, no significant difference was observed in fluorescent signal measured in the lung-draining mLNs of Cre(+) vs Cre(−) YAP/TAZ^△LEC^ littermates at baseline or at 7dpi (Fig. 5i and j). Collectively, the targeted deletion of YAP and TAZ in LECs did not affect lymphangiogenesis or lung lymphatic drainage at baseline or during influenza pneumonia.

### Infection severity and inflammatory response after deletion of YAP and TAZ in LECs.

**Figure 6:**
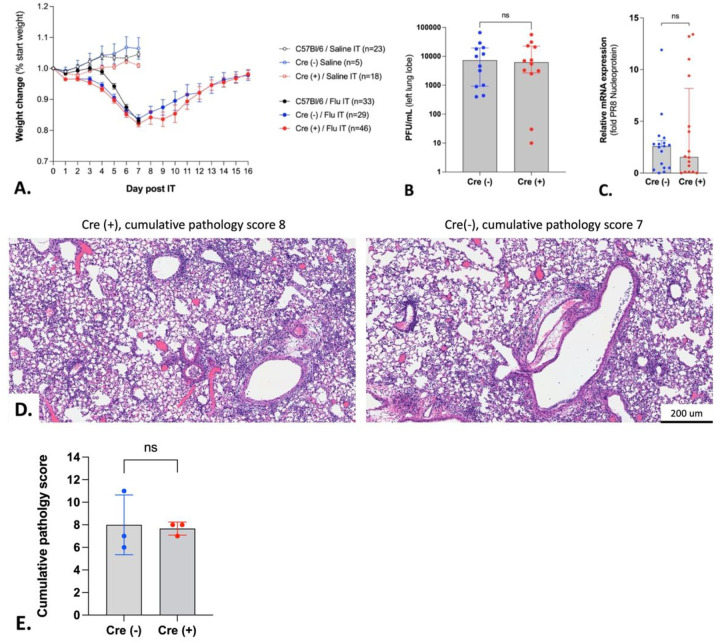
There is no significant difference in infection severity between Cre(−) and Cre(+) YAP/TAZ^△LEC^ littermates after influenza challenge. (A) Weight change for C57Bl/6 mice and Cre(−) and Cre(+) YAP/TAZ^△LEC^ littermates after saline or influenza challenge. Mean and SEM are shown. (B) Influenza PFU recovered from left lobes of Cre(−) or Cre(+) littermates at 7 dpi after influenza challenge. Median and IQR are shown for n = 12 mice per group. Statistical significance by unpaired two-tailed Mann Whitney test. (C) Nucleoprotein (NP) mRNA was quantified from left lobes collected from Cre(−) or Cre(+) littermates at 7 dpi after influenza infection. Median and IQR are shown for n = 16–17 mice per group. Statistical significance by unpaired two-tailed Mann Whitney test. (D–E) Histopathological scoring by a blinded veterinary pathologist was performed using H&E-stained lung sections from Cre(−) or Cre(+) littermates at 7 dpi after influenza infection; representative sections are shown. Mean and SD are shown for n=3 mice per group. Statistical significance by unpaired two-tailed Mann Whitney test.

Infection severity (28) and inflammatory responses (9, 29) are known to be affected by aberrant lymphatic function. To address this issue, we first compared survival and weight loss curves after influenza pneumonia in Cre(+) and Cre(−) YAP/TAZ^△LEC^ littermates. No mice reached the humane endpoint in either group, and we found no differences in weight change during or after influenza infection between the two Cre genotypes (Fig. 6a). We then assessed infection severity by plaque assay and qRTPCR for influenza nucleoprotein *(NP)* mRNA in murine lungs at 7 dpi and found no difference in these readouts between Cre genotypes (Fig. 6b and c). We also characterized the inflammatory response to influenza in the lung at 7 and 16 dpi in both Cre genotypes. These timepoints mark two critical phases: the lowest point of weight loss and the subsequent return to baseline weight after influenza infection. Detailed inflammation scoring and histopathologic characterization was provided by a veterinary pathologist who was blinded to genotypes. In summary, there were no apparent differences due to YAP/TAZ-deletion in morphological histopathology as assessed by the percent area of consolidation or semiquantitative pathology score (Fig. 6d and e, Supp. Tables 1 and 2).

**Figure 7:**
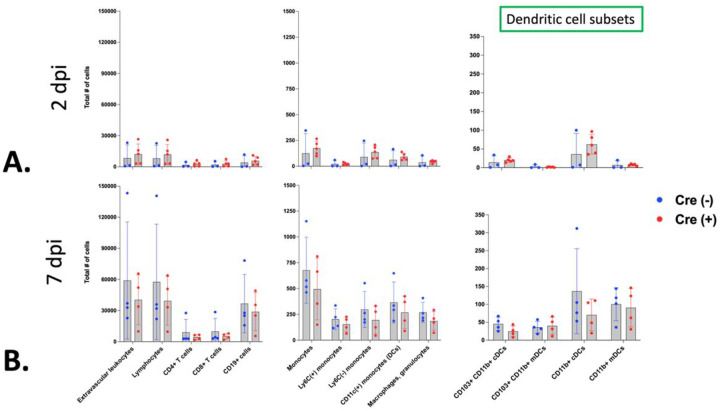
There is no significant difference in mLN immunophenotype between Cre(−) and Cre(+) YAP/TAZ^△LEC^ littermates after influenza challenge. An 11-color flow cytometry panel was used to determine the immunophenotype of the mLN of Cre(−) or Cre(+) littermates at 2 dpi (A) and 7 dpi (B). After the exclusion of doublets and debris, dead cells were excluded using live/dead staining. Immune cells were identified using the pan-hematopoietic marker CD45 (including separate stains for intravascular vs tissue resident CD45(+) cells). The panel included markers for T- cells (CD4, CD8), B-cells (CD19) as well as dendritic cell subtypes (Ly6C, CD11b and CD103 positive and negative subsets of CD11c(+) cells). Monocytes, macrophages and granulocytes were classified generally using Ly6C, CD64, Ly6G and Siglec F. Mean and SD are shown for n = 3–5 mice per group per timepoint.

Lastly, to evaluate differences in immune cell transit between the lung and the lung-draining mLN, we designed an 11-color flow cytometry panel to determine the immunophenotype of the lymph node at 2 and 7 dpi. Using markers for T- cells (CD4, CD8) and B-cells (CD19) as well as markers for dendritic cell subtypes (Ly6C, CD11c, CD11b and CD103), we were able to define cell populations known to be important in the developing adaptive immune response, for which a functional lymphatic vasculature is known to be crucial (28, 30). While we did observe predictable differences in the mLN immunophenotype between 2 and 7 dpi, we identified no differences in any of the defined immune cell populations between Cre(+) or Cre(−) YAP/TAZ^△LEC^ littermates (Fig. 7a and b).

## Discussion

Our studies demonstrate that the pulmonary lymphatic system expands significantly during severe influenza, via both dilation and lymphangiogenesis. This is a stark change from the quiescent state of the lymphatic plexus during homeostasis (31) and is reminiscent of the rapid lymphatic growth seen in development (32). Furthermore, our findings demonstrate that lymphangiogenesis occurs primarily through proliferation of existing LECs, without an apparent involvement of an exogenous precursor. Moreover, we found that following this expansion, the lymphatic vasculature displays enhanced fluid transport.

The inflammation resulting from influenza infection may trigger responses in LECs through mechanical signals. For example, alterations in extracellular matrix (ECM) composition may modulate matrix stiffness, as has been reported in pulmonary fibrosis (33). Higher fluid volume within the lymphatics can increase circumferential stretch (34). As YAP/TAZ intracellular localization is sensitive to such physical changes (35–37), we investigated whether the Hippo pathway plays a part in orchestrating the lymphatic response to influenza-induced inflammation.

We found that during influenza-induced lymphangiogenesis, the targeted deletion of YAP and TAZ in PROX1-expressing cells had no effect on fluid transport from the lung to the draining lymph nodes at baseline or during influenza infection. Lymphatic vessel diameter, LEC number, *Prox1* and *Flt4* mRNA transcription and lung wet-to-dry weight ratios at 7 dpi were also unaffected by LEC-specific YAP/TAZ deletion.

Previous work has yielded conflicting findings regarding the regulatory relationship between YAP/TAZ and PROX1. Using PROX1-driven, Cre-mediated deletion of YAP and TAZ, Cho *et. al*. find that YAP and TAZ activity opposes PROX1 expression during development, limiting lymphangiogenesis (23). Conversely, in vitro experiments show that both YAP and TAZ knockdown and hyperactivation are associated with diminished PROX1 expression (24). In our studies, the lymphatic remodeling we observed in response to influenza was not perturbed or enhanced in YAP/TAZ^△LEC^ animals. In this regard, deleting these effectors did not significantly affect the morphological or functional characteristics measured. While Hippo signaling is involved in fundamental cellular events during both development and in tissue repair in the adult (17), our results show that YAP and TAZ do not play a role in lung LECs during the lymphangiogenic response to influenza. In this respect, the role of Hippo signaling during lung lymphangiogenesis in adulthood contrasts with its apparent function during development. Overall, our work demonstrates that during inflammation-induced expansion of pre-existing lymphatic vasculature during adulthood, Hippo signaling is dispensable in LECs.

Broadly speaking, it is important to note that the precise role of an expanded lymphatic network accompanying tissue injury remains controversial and possibly organ and context-dependent (9, 10, 12, 13, 27, 38–41). In tracheal *Mycoplasma* pulmonis infection, inhibiting lymphangiogenesis promotes edema and interferes with immune cell trafficking to lymph nodes (10), while in a mouse model of pulmonary fibrosis, promoting lymphangiogenesis was shown to reduce type I collagen accumulation (12). Further, in a mouse model of aspiration pneumonia, inhibition of lymphatic growth improved oxygen saturation (39). However, inducing lymphangiogenesis reduced inflammation, improved lymphatic drainage and increased left lung aeration in mouse lung transplant allografts (27). In the skin, lymphangiogenesis facilitates tissue resilience (9, 40). Most notably, genetic blockade of lymphangiogenesis did not significantly affect cardiac function in a mouse model of myocardial infarction (41). Whether an expanded lymphatic network facilitates tissue recovery from diffuse lung injury will require additional study. This study demonstrates that Hippo signaling is not critical for lymphatic function and expansion during influenza. The question as to whether the expanded network is required for recovery remains to be answered.

## Materials and Methods

### Mice

C57BL/6J mice were obtained from Jackson laboratories and housed in the Boston University Animal Science Center on a 12-hour light-dark cycle with access to food and water *ad libitum*. Prox1-Cre-ER^T2^ mice and TdTomato reporter mice were obtained from The Jackson Laboratory (strain #022075 and strain #007914, respectively). The YAP^loxP/loxP^ mice and Wwtr1/TAZ^loxP/loxP^ mice used to generate the YAP/TAZ^△LEC^ mice reported here were provided by Dr. Jeffrey Wrana (LTRI institute) (Jackson Laboratory Strain #030532). Our study examined both male and female animals with the sex of the mice randomized across experimental groups. Similar findings are reported for both sexes. At time of sacrifice, mice were ethically euthanized using isoflurane and inferior vena cava incision. All methods were carried out in accordance with relevant IACUC guidelines and regulations and are reported in accordance with ARRIVE guidelines. All animal experiments were performed in compliance with approved Boston University animal protocols PROTO201800710_TR01 and PROTO201800057_TR01.

### Influenza Infection

Mice aged 8–16 weeks were anesthetized via intraperitoneal injection of 75 mg/kg ketamine and 10 mg/kg xylazine and infected with 20–400 PFU of influenza A/H1N1/Puerto Rico/8/34 (PR8) virus via intratracheal instillation directed into the left lung lobe. Mice were weighed daily post-infection until time of sacrifice or until all animals had returned to their pre-infection weights. In all studies, a humane endpoint of reaching 70% of starting body weight was employed.

### Tamoxifen administration

To induce Cre activity, Prox1-Cre-ER^T2^ mice aged 6–12 weeks were administered 100 mg/kg tamoxifen dissolved in corn oil to a concentration of 30 mg/mL via intra-peritoneal injection daily for 5 days, then rested for at least 14 days before experimental use.

### EdU administration

To induce EdU labeling of proliferating cells, influenza infected or control C57BL/6J mice aged 8–12 weeks were administered 10 mg/kg EdU dissolved in sterile saline to a concentration of 2.5 mg/mL via intra-peritoneal injection daily from 4 to 6 dpi.

### Tissue Processing

Mice were euthanized and their lungs perfused through the left ventricle of the heart using 10 mL of ice-cold HBSS. Left lungs were fixed overnight at 4º C in 4% paraformaldehyde, then washed in PBS (pH 7.4), dehydrated and embedded in paraffin. Paraffin embedded lungs were cut into 5 μm sections using a microtome and dried overnight at 42º C.

### Immunostaining and microscopy

Paraffin-embedded tissue sections were deparaffinized in xylene and then rehydrated. Antigen- retrieval was performed by heating with citrate-based antigen-unmasking solution (Vector). For experiments using HRP detection, endogenous peroxidase was quenched using 3% hydrogen peroxide in methanol. Nonspecific antigen binding was blocked using donkey serum. Sections were incubated with primary antibodies at the dilutions shown below before application of secondary antibodies. Samples containing EdU were also counter stained per protocol using the Click-IT^™^ EdU Cell Proliferation Kit (Invitrogen #C10339). For HRP detection, Vectastain ABC and DAB kits were used. Sections were counterstained using hematoxylin, dehydrated and cover slipped. Samples using immunofluorescent antibodies were counter-stained with DAPI before analysis.

For PRoX1 nuclei counting, non-serial murine lung tissue sections approximately 350 μm apart were stained as described above. Nuclei were visualized via light microscopy using a Nikon Eclipse E200 microscope or Leica DM4 B microscope with a Leica DFC7000 T camera. Immunofluorescent samples were imaged using a Leica DM4 B microscope with a Leica DFC7000 T camera. We would also like to acknowledge S10oD030269 instrumentation for supporting the microscopic analysis. See Table 1 for list of antibodies and dilutions.

### Histopathology scoring and quantitative image analysis

Digitalized whole slide images (WSI) of brightfield H&E were generated using PhenoImager HT 2.0 Automated Quantitative Pathology Image System (Akoya Biosciences, Marlborough, Massachusetts, USA). H&E-stained slides were scanned at a 200x magnification using a bright field scan profile. Quantification of the pulmonary inflammatory lesions were achieved by using the HALo image analysis software v3.5 (Indica labs). Each image was first annotated by a veterinary pathologist to define the regions of interest for image analysis. Total examined pulmonary parenchyma with associated airways of three replicate sections were included in the region of interest. All processing artifacts (i.e. tissue folds and dust) were also manually removed via exclusion annotations. Using the random forest V2 algorithm provided with HALo image analysis software, two classes were defined: normal and pulmonary consolidation. The full spectrum of histopathological changes was included in the algorithm, which included lymphoplasmacytic and histiocytic interstitial pneumonia and type 2 pneumocyte hyperplasia and dysplasia. The exact same classifier algorithm was applied to all images in this study to yield the pulmonary consolidation quantification results. The pathologist reviewed all the slides in a blinded fashion and developed ordinal histopathology criteria for scoring (Supplemental Tables 1 and 2).

For quantitative image analysis of YAP and TAZ immunofluorescent staining in LECs in lung histologic sections, PRoX1 staining was used to localize the cells of interest and median fluorescence intensity (MFI) of either YAP or TAZ staining was quantified using CellProfiler^R^. Data were obtained by imaging all LECs on at least 3 separate non-serial histologic lung sections per mouse.

### Flow cytometry

Mice were anesthetized via intraperitoneal injection of 75 mg/kg ketamine and 10 mg/kg xylazine and injected retro-orbitally with 2 ug of IV CD45.2 antibody. This was allowed to circulate for 3 minutes prior to sacrifice. Mediastinal lymph nodes (mLNs) were collected from influenza infected mice as well as controls and placed in 1 mL of cold PBS. LN tissue was passed through a 100 um strainer in a petri dish by crushing with a rubber syringe plunger and the strainer was washed with 3 mL cold FACS buffer. The single cell suspension was placed in 15 mL FACS tubes and centrifuged at 300xg at 4º C for 5 minutes, and the cell pellet was resuspended in 2 mL FACS buffer. Cell number was enumerated using a hemocytometer. Cell suspensions were diluted to 1×10^6^ cells/mL and 1 mL of cell suspension (1×10^6^ cells) placed in new FACS tubes. Cell solutions were blocked with anti-CD16/32 (FcBlock, eBioscience) at a dilution of 1 ug per 1×10^6^ cells. Fluorochrome-conjugated monoclonal antibody staining was performed using the antibodies and dilutions noted in the table for 30 minutes on ice in the dark. Excess antibody was removed by washing with 1 mL FACS buffer and centrifugation at 300xg at 4ºC for 5 minutes. The cell pellet was resuspended in 300 μL FACS buffer. Cells were stained with 7-AAD Viability Staining Solution (BD Biosci.). Unstained cells, single-stained oneComp eBeads (eBioscience), and fluorescence-minus-one (FMo) controls were used for each analysis. Data were acquired on a BD LSR II flow cytometer (BD Biosciences) using BD FACSDiva software. FlowJo^R^ 10 was utilized for data analysis. See Table 2 for list of antibodies and dilutions.

### qRT-PCR

RNA isolation from mouse whole lung homogenates was performed using the Direct-zol RNA Microprep kit (Zymo Research) according to the manufacturer’s instructions. qRT-PCR was performed using the Taqman and QuantStudio 3 (Applied Biosystems) systems according to the manufacturer’s instructions. See Table 3 for list of primers and probes.

### Viral plaque assay

Left lung lobes from influenza infected mice were minced using a sterile razor blade in a petri dish, transferred to a bullet blender tube in 2 mL of viral growth media (as below), and homogenized. Cell debris was pelleted by centrifugation at 5,000xg at 4ºC for 5 minutes, and supernatant was collected and used to infect MDCK cells. Plaques were enumerated after 3 days (42).

Viral growth media (500mL):

- 475mL of DMEM (ThermoFisher ref 11995–065)
- 5mL Pen/Strep 100x (ThermoFisher 15140122)
- 12.5mL of 1M HEPES (Sigma-Aldrich H0887)
- 5mL Glutamax 100x (ThermoFisher 35050061)
- 3mL of 35% BSA stock (Sigma-Aldrich A7409) oR 3.3ml of 30% BSA

### Lung wet to dry weight ratio

Left lung lobes were collected from influenza infected and uninfected mice. Lungs were weighed immediately after collection to obtain the wet weight, then desiccated for 48 hours at 65ºC and weighed again to obtain the dry weight (43).

### Dextran transport assay

Mice were anesthetized via intraperitoneal injection of 75 mg/kg ketamine and 10 mg/kg xylazine before injection intratracheally into the left lung lobe with 10 μL of 10 mg/mL of 10kDa Dextran-488 (Invitrogen #D22910) dissolved in sterile normal saline (NS), or equivalent volume of NS (vehicle-only) for control mice (44). Mice were allowed to recover for 15 or 50 minutes prior to sacrifice. Mediastinal LNs and inguinal LNs were collected from each mouse and placed in 280 μL of protein extraction buffer (EB; Tris pH 7.4 25mM, NaCl 50mM, 0.5% Na deoxycholate, 2% NP-40, and 0.2% SDS dissolved in ddH2o) with proteinase inhibitor (Roche #11836153001, final 1x concentration). LN tissue was passed through a 70 μm strainer in a petri dish by crushing with a rubber syringe plunger and the strainer was washed with 200μL cold EB. Cell debris was pelleted by centrifugation at 15,000xg at 4ºC for 20 minutes, and supernatant was collected. Fluorometric quantification was performed using an Agilent BioTek Synergy LX Multi-Mode fluorescence plate reader. Fluorometric values for mLNs were obtained by subtracting background fluorescence as measured in inguinal LNs.

### Statistical analysis

Statistical significance was determined using a two-tailed Student’s t-test in the case of data shown to be parametric via the Shapiro-Wilk test. Welches’ correction was used to account for differences in variance. If the data was non-parametric, a Mann-Whitney U test was used. A *p*-value of 0.05 was used as the threshold for statistical significance.

## Figures and Tables

**Figure 3:** LECs proliferate in response to influenza infection. (A) C57Bl/6 mice were administered saline or PR8 followed by EdU injections, and lungs were harvested at 7 dpi followed by IF staining for PRoX1 and EdU. White arrowheads indicate PRoX1+/EdU+ cells. (B) The number of PRoX1+ LECs that costained for EdU was quantified; mean and SD are shown for n = 9 mice per condition. Statistical significance by unpaired two-tailed Mann Whitney test. (C) Prox1-CreER^T2^ / TdTomato mice were administered tamoxifen prior to saline or PR8 administration, and lungs were harvested at 7 dpi followed by IF staining for PRoX1 and tdTomato. White arrowheads indicate PRoX1+/TdTomato+ cells. (D) The number of PRoX1+ LECs that costained for tdTomato was quantified; mean and SD are shown for n = 5 mice per condition. Statistical significance by unpaired two-tailed Mann Whitney test.

**Table 1:** List of antibodies used for histologic staining.

Antibody	Application	Source	Catalog #	Dilution
Rabbit -PROX1 (EPR19273)	Immunostaining	Abcam	ab199359	1:500 for immunostaining
Goat -VEGFR3	Immunostaining	R&D Systems	AF743	1:500
Rabbit -TAZ (E8E9G)	Immunostaining	Cell Signaling Technologies	83669	1:100
Rabbit -YAP (D8H1X, XP)	Immunostaining	Cell Signaling Technologies	14074	1:100
Goat -PROX1	Immunostaining	R&D Systems	AF2727	1:300 in conjunction with YAP/TAZ staining

**Table 2:** List of antibodies used for flow cytometry.

Cell types	Target	Fluorophore	Concentration / Dilution	Source and Catalog #
Live / dead	7-AAD	7-AAD	1:60	BD Biosci. 51-68981e
Leukocytes	CD45 (clone 30-F11)	Per-CP 5.5	0.2 mg/mL	Biolegend 103131
	CD45.2 (IV) (clone 104)	BUV737	0.2 mg/mL	BD Biosci. 612778
T cells	CD4	APC-Cy7	0.2 mg/mL	Biolegend 100413
	CD8a	BV510	0.05 mg/mL	Biolegend 100751
B cells	CD19	BUV395	0.2 mg/mL	BD Biosci. 563557
Dendritic cells	CD11 b	PE	0.2 mg/mL	Biolegend 101207
	CD11c	APC	0.2 mg/mL	Invitrogen 17-0114-81
	CD103 (clone 2E7)	PE-Cy7	0.2 mg/mL	Biolegend 121425
	Ly6c	Efluor450	0.2 mg/mL	Invitrogen 48-5932-80/2
Dump gate	CD64	FITC	0.5 mg/mL	Biolegend 161007
	Ly6g	FITC	0.5 mg/mL	Biolegend 127606
	Siglec F	FITC	0.5 mg/mL	Biolegend 155503

**Table 3:** List of primers and probes used for PCR and qRT-PCR.

Target	Primer / Probe	Source and Catalog #
Prox1	Mm00435969_m1	TaqMan^R^ 4331182
Flt4	Mm01292604_m1	TaqMan^R^ 4331182
Yap1	Mm00494240_m1	TaqMan^R^ 4331182
	F: AATCGAGAAACCGCTGGGG (sense)	IDT DNA (custom)
	R: GGAGGCCAAACCTGACAACTA (antisense)	IDT DNA (custom)
Taz	Mm00504978_m1	TaqMan^R^ 4331182
	F: GACATTCCAATCCTCCCACTAC (sense)	IDT DNA (custom)
	R: GGT CCAG CCCATAACTACTTTAC (antisense)	IDT DNA (custom)
Influenza Nucleoprotein (NP)	PR8seg5-F: 5’-CGT TCT CCATCAGTCTCCATC- 3’	IDT DNA (custom)
	PR8seg5-R: 5’-GAGT GACAT CAAAAT CAT GGCG-3’	
	Probe: 5’-AG G CACCAAACG G T CTT ACGAACA-3’	

## Data Availability

The datasets generated during and/or analyzed during the current study are available from the corresponding author on reasonable reque
